# Wilms Tumor with Inferior Vena Cava Thrombus: Comparative Analysis of Clinical Characteristics and Outcomes

**DOI:** 10.3390/curroncol33040197

**Published:** 2026-03-31

**Authors:** Rahaf Al-Hasan, Rula Al-Qawabah, Khalil Ghandour, Ahmad Kh. Ibrahimi, Nasim Sarhan, Iyad Sultan, Hadeel Halalsheh

**Affiliations:** 1 Department of Pediatrics, King Hussein Cancer Center, Amman 11941, Jordan; ra.17346@khcc.jo (R.A.-H.); isultan@khcc.jo (I.S.); 2 Department of Radiology, King Hussein Cancer Center, Amman 11941, Jordan; ra.14467@khcc.jo; 3 Department of Pediatric Surgery, King Hussein Cancer Center, Amman 11941, Jordan; jafapal48@yahoo.com; 4 Department of Radiation Oncology, King Hussein Cancer Center, Amman 11941, Jordan; aibrahimi@khcc.jo (A.K.I.); nsarhan@khcc.jo (N.S.); 5 Artificial Intelligence Office, King Hussein Cancer Center, Amman 11941, Jordan; 6 Department of Pediatrics, The University of Jordan, Amman 11941, Jordan

**Keywords:** Wilms tumor, outcome, IVC, inferior vena cava, thrombus

## Abstract

Background: An inferior vena cava thrombus (IVCT) occurs in approximately 4–10% of Wilms tumor (WT) cases. The presence of an IVCT complicates surgical management and potentially affects patient outcomes. We reviewed all patients with a WT with and without an IVCT treated at our center, comparing their characteristics and outcome. Patients and Methods: We retrospectively reviewed pediatric patients with a WT treated between November 2014 and July 2023. Patients were categorized according to the presence or absence of an IVCT. Data on demographics, clinical presentation, imaging findings, treatment modalities, and outcomes were collected. Group comparisons for categorical data were carried out using Chi-square tests, Event-free survival (EFS) and overall survival (OS) were estimated using Kaplan–Meier curves, and differences between groups were assessed with the log-rank test. Results: Among 110 children with a unilateral WT, 17 (15.4%) had an IVCT at presentation. Their median age at diagnosis was 4.2 years. Females predominated (65%) and 11 (65%) had distant metastasis. Thrombus levels were infra-hepatic (52.9%), retro-hepatic (23.6%), supra-hepatic (5.9%), and intra-cardiac (17.6%). Thrombus resolution following neo-adjuvant chemotherapy was achieved in 41.2% of patients, most frequently in infra-hepatic thrombi. Local control followed neo-adjuvant chemotherapy that included doxorubicin. Favorable histology was observed in 70% of patients with an IVCT. Compared with patients without an IVCT, children with an IVCT tend to present at an older age, have hematuria, a larger tumor, positive lymph node (LN) involvement, and metastatic disease (*p* value, 0.019, 0.008, 0.01, 0.014, and 0.002, respectively). On univariable analysis, older age, the presence of metastatic disease, IVCT, LN involvement, and diffuse anaplasia were associated with inferior EFS and OS. Five-year EFS and OS were significantly lower in patients with IVCT compared to those without (51% ± 12.5% vs. 74.5% ± 4.6%, *p* = 0.021 and 53.8% ± 15.4% vs. 82% ± 4.9%, *p* = 0.012, respectively). Conclusions: We observed a higher proportion of IVCT than previously reported. Consistent with prior studies, an IVCT was associated with more aggressive disease features. Although survival was worse in patients with an IVCT, the mere presence of a thrombus as an independent prognostic factor requires evaluation in larger patient cohorts.

## Simple Summary

Wilms tumor is the most common pediatric kidney malignancy, and in a subset of patients, the tumor can extend into the inferior vena cava, complicating surgical management. In this study, we compared clinical characteristics and outcomes of children with and without vascular involvement. Patients with a thrombus presented with larger tumors, more frequent lymph node involvement, and higher rates of metastatic disease. While survival was lower in this group, the presence of a thrombus as independently predictive of outcome needs to be studied in a larger patient cohort. These findings underscore the importance of risk-adapted, multidisciplinary management and provide contemporary evidence to guide surgical planning, therapeutic decisions, and future research for high-risk Wilms tumor patients.

## 1. Introduction

Wilms tumor (WT) is the most common primary renal malignancy of childhood, accounting for approximately 5–7% of all pediatric cancers and is often diagnosed before the age of five years [1,2]. Over the past several decades, major advances in multimodal treatment, including risk-adapted chemotherapy, surgery, and radiotherapy, have resulted in excellent outcomes, with overall survival now exceeding 90% in recent cooperative group studies [3]. Nevertheless, a subset of patients continues to experience inferior outcomes, particularly those presenting with high-risk disease features [4].

One such high-risk feature is tumor extension into the renal vein and inferior vena cava [5]. Inferior vena cava tumor thrombus (IVCT) formation occurs in approximately 4–10% of children with Wilms tumor and represents a distinct clinical entity associated with increased surgical complexity and risk of perioperative morbidity [6,7,8]. In rare cases, the tumor thrombus may extend into the right atrium, further complicating management and necessitating cardiopulmonary bypass during resection [7,8,9].

Historically, the presence of intravascular extension was considered an adverse prognostic factor; however, recent studies have shown conflicting results regarding its independent impact on survival [5,10]. While some reports demonstrate comparable outcomes between patients with and without an IVCT when treated with multimodal therapy, others suggest an association with advanced stage, metastatic disease, and inferior survival [5,7]. These discrepancies may reflect differences in patient populations, tumor biology, treatment protocols, and the extent of vascular involvement.

Modern management strategies for WT with an IVC thrombus often include neo-adjuvant chemotherapy to reduce tumor burden and facilitate safer surgical resection, particularly in cases with supra-diaphragmatic extension [11,12,13,14]. The role of radiotherapy and the prognostic implications of residual thrombus, addition of doxorubicin, and histologic subtype in patients with IVCT continue to be areas of active investigation [15,16,17].

Given the relative rarity of this presentation and the limited data from low- and middle-income countries, and the potential impact of resource availability, surgical expertise, and treatment adherence on outcomes, further characterization of clinical features, treatment approaches, and outcomes from single-center experiences remains essential. In this study, we describe our institutional experience in patients with WT, stratified by the presence or absence of an IVCT, and compare their characteristics and outcomes.

## 2. Patients and Methods

Following approval from the King Hussein Cancer Center (KHCC) institutional review board (IRB-25 KHCC 059), we conducted a retrospective review of medical records of pediatric patients diagnosed with WT treated at our institution between November 2014 and July 2023. Follow-up data were described through December 2024. We excluded patients who presented for consultation, radiation therapy, or surgery only. Patients were divided into two groups based on the presence or absence of IVCT at diagnosis. We compared patient characteristics and outcomes between those with IVCT and those without.

We collected demographic data, including age at diagnosis, sex, as well as clinical data including symptoms and signs at presentation, imaging findings (laterality, metastasis, and presence of IVCT, extent of vascular involvement at presentation and after neo-adjuvant chemotherapy), stage distribution, tumor histology, time of surgery whether upfront or after neo-adjuvant chemotherapy, chemotherapy regimens, and radiation therapy including dose and site.

Staging and chemotherapy protocols were based on International Society of Pediatric Oncology (SIOP) 2001 (before 2017) and UMBRELLA (2017 and beyond). As our institution serves as a referral cancer center, a proportion of patients were referred after upfront nephrectomy performed at outside hospitals. In these cases, management followed the specific post-nephrectomy staging and risk-adapted treatment recommendations provided within the SIOP protocols. Patients with localized disease received four weeks of vincristine and actinomycin (VA), while patients with metastasis at diagnosis received six weeks of VA and doxorubicin. Chemotherapy regimen following local surgical control was based on post-treatment pathology, stage, and tumor volume.

All imaging studies were retrospectively reviewed by experienced pediatric radiologist (R.Q). Contrast-enhanced computed tomography (CT) and/or Magnetic Resonance Imaging (MRI) scans obtained at diagnosis and after completion of neo-adjuvant chemotherapy were evaluated to assess the presence and extent of intravascular tumor thrombus. IVCT was defined radiologically as tumor extension into the inferior vena cava, with or without extension into the hepatic veins or right atrium. Patients with tumor thrombus confined to the renal vein (without extension into the IVC) were classified in the non-IVCT group. The cranial (cephalic) extent of the IVC tumor thrombus was classified based on its anatomic relationship to the hepatic veins and IVC as follows: Level I (infra-hepatic), thrombus extending into the IVC below the hepatic veins; Level II (retro-hepatic), thrombus extending into the IVC at the level of the hepatic veins; Level III (supra-hepatic), thrombus extending into the IVC above the hepatic veins but below diaphragm; and Level IV (intra-cardiac), thrombus extending into the right atrium. Baseline and post neo-adjuvant chemotherapy imaging were compared to assess changes in thrombus extent, with regression, stability, or progression recorded accordingly.

Overall survival (OS) was calculated from the time of diagnosis to death or the most recent follow-up for patients still alive. Event-free survival (EFS) was calculated from diagnosis to first relapse, progression, or death from any cause, or to the last follow-up for patients who remained event-free.

Cases underwent comprehensive review by the multidisciplinary team (MDT) at predefined intervals during the treatment course, including at the time of diagnosis, upon achievement of local control, postoperatively following receipt of the pathology report, and in the event of any significant change or complication. The MDT included a team of medical professionals, including a pediatric oncologist, pediatric surgeon, pathologist, radiologist, and radiation oncologist. This strategy allowed for personalized treatment adjustments, including optimized timing of surgery, modified chemotherapy protocols, and targeted radiation to either the primary tumor or metastatic sites.

## 3. Statistical Analysis

Patient demographics, tumor features, and treatment details were described using descriptive statistics. Continuous data were reported as medians with an interquartile range (IQR), and categorical data as counts and percentages. Between-group comparisons for categorical variables used Chi-square or Fisher’s exact tests, and differences in continuous variables were assessed using Student’s *t*-tests or non-parametric alternatives (Mann–Whitney U tests) depending on data distribution. Univariable and multivariable Cox regression models were performed to identify factors independently associated with EFS and OS. The multivariable model adjusted for key confounders such as age, sex, cancer stage, and treatment modality. OS and EFS were estimated using the Kaplan–Meier method, and survival curves were compared with the log-rank test where appropriate.

Univariable Cox proportional hazards regression was performed to evaluate associations between clinical and pathological factors and survival outcomes; results are expressed as hazard ratios (HRs) with 95% confidence intervals. Model fit is characterized for each covariate by the Nagelkerke pseudo R^2^ and Harrell’s C-statistic (concordance index).

Multivariable Cox regression was precluded because the EPV ratios for EFS and OS did not meet the minimum threshold of 10 required for robust inference. Utilizing a multivariable model in this context risks severe overfitting, biased hazard estimates, and falsely precise confidence intervals.

All analyses were performed in R version 4.4.3 (R Core Team, Vienna, Austria); statistical significance was defined as a two-sided *p*-value < 0.05.

## 4. Results

### 4.1. Patient Characteristics

A total of 110 pediatric patients were diagnosed with a WT between November 2014 and July 2023 at KHCC, and met the eligibility criteria for the study. IVCT was identified in 17 patients (15.4%) at diagnosis, while 93 patients (84.5%) had no IVC involvement.

Patients with an IVCT were slightly older at diagnosis, with a median age of 4.2 years (range: 2.6–15.1) compared with 3.5 years (range: 0.4–11.3) in patients without thrombosis (*p* = 0.019). Sex distribution was comparable between groups; overall, 56% of patients were female, Table 1.

Hematuria was significantly more frequent among patients with an IVCT (47% vs. 16%, *p* = 0.008). Tumor laterality showed significance (*p* = 0.046). The IVCT group exhibited a higher proportion of right-sided tumors (65%), whereas left-sided tumors were more prevalent (61%) in patients without evidence of IVC involvement.

The presence of an IVC tumor thrombus (IVCT) was significantly associated with more advanced disease stages. Stage IV disease occurred in 65% of IVCT patients versus 26% in patients without IVC involvement (*p* < 0.001), predominantly manifesting as pulmonary metastases. Lymph node involvement was also more frequent in the IVCT group (41% vs. 14%, *p* = 0.014). Patients with an IVCT had significantly larger tumors (median size: 12.7 cm vs. 11.9 cm, *p* = 0.01)

Overall, abdominal mass was the most common presenting symptom (75%) in both groups. Other presenting features, including abdominal pain and hypertension did not differ significantly between groups.

### 4.2. Anatomical Extent and Response of IVCT to Neo-Adjuvant Therapy

Among the 17 patients presenting with an IVC tumor thrombus, the infra-hepatic thrombus location predominated at diagnosis. Intra-cardiac extension was observed in three patients (17.6%), retro-hepatic thrombosis in two (23.6%), and supra-hepatic involvement in one (5.9%).

Following neo-adjuvant chemotherapy and prior to surgical intervention, complete radiological resolution of the inferior vena cava tumor thrombus (IVCT) was observed in 41.2% (*n* = 7) of patients. Resolution rates differed according to the initial thrombus level: infra-hepatic thrombi demonstrated the greatest propensity for resolution (55.6%; 5/9), followed by retro-hepatic thrombi (50%; 2/4). In contrast, complete resolution was not achieved in any cases of intra-cardiac or supra-hepatic involvement, with all such patients requiring surgical thrombectomy due to persistent thrombus (Table 2).

### 4.3. Treatment Modalities

Most patients completed surgical local control following neo-adjuvant chemotherapy (85.5%), with no statistically significant difference between patients with and without an IVCT (100% vs. 83%, *p* = 0.13). Upfront surgery was performed in 14% of patients, all of whom belonged to the non-IVCT group.

All patients ultimately underwent radical nephrectomy with LN sampling. Surgical thrombectomy was ultimately required in 10 of the 17 patients (59%) with an IVC tumor thrombus (IVCT). Technique of thrombus extraction was either through the renal vein (*n* = 4, typically for lower-level or resolved-adjacent thrombi) or necessitated a limited cavotomy (*n* = 6, for adherent or higher-level involvement). None of the patients required a cardiopulmonary bypass. Among the seven individuals in whom preoperative imaging demonstrated complete radiological resolution of the IVC tumor thrombus, all except one exhibited no identifiable thrombus intraoperatively or upon pathological analysis. In one patient, however, a thrombus was detected intraoperatively in the infra-hepatic IVC, which was successfully removed and confirmed on pathological evaluation.

Among the remaining patients with radiologic evidence of persistent thrombus, one case showed an infra-hepatic IVC thrombus on preoperative imaging, yet no macroscopic thrombus was identified intraoperatively; however, pathological examination confirmed microscopic viable tumor cells in the renal vein with negative margins.

Perioperative complications occurred in 23.5% the IVCT group: lymphatic leak (*n* = 2), removal of part of the diaphragm densely attached to the tumor (*n* = 1), and inadvertent long IVC tear (*n* = 1), though there was no perioperative mortality.

Chemotherapy regimens differed significantly between groups (*p* < 0.001). All patients with an IVCT received a doxorubicin-containing regimen.

Radiotherapy was significantly more common in the IVCT group, with a median dose of 14.4 Gy (range, 0–36) compared with a median dose of 0 Gy in patients without thrombosis (*p* < 0.001). Among patients with an IVCT, 65% received flank radiation and 24% received whole-abdominal radiation, while 68% of patients without thrombosis did not receive any radiotherapy.

### 4.4. Outcomes and Survival Analysis

**Figure 1:**
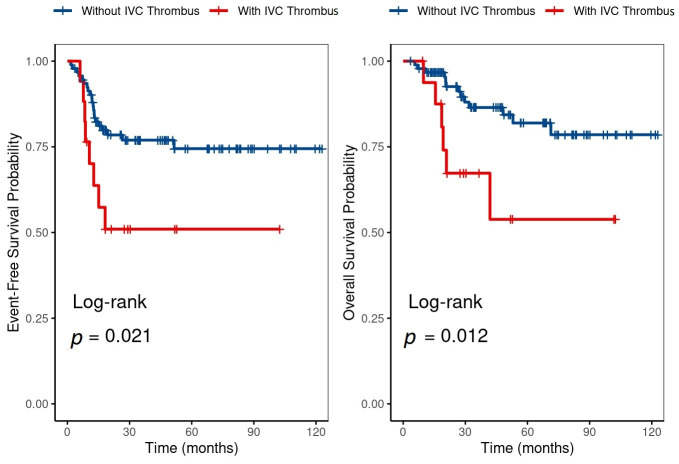
Kaplan–Meier event-free survival and overall survival curves for patients with and without inferior vena cava thrombus (IVCT).

During a median follow-up of 5 years (range: 2–9 years), both 5-year EFS and OS were significantly lower in the IVCT group compared to those without (51 ± 12.5% vs. 74.5 ± 5.1%, *p* = 0.021, and 53.8 ± 15.4% vs. 82 ± 4.9%, *p* = 0.012, respectively), Figure 1.

The univariable Cox regression analysis for EFS identified several significant prognostic factors. Metastasis was the strongest predictor of poor outcomes, associated with a nearly four-fold increase in risk (HR: 3.86; 95% CI: 1.84–8.11; *p* < 0.00) and providing the highest model discrimination (C-statistic = 0.67) and explanatory power (Nagelkerke R^2^ = 0.11). Other significant factors included diffuse anaplasia (DA) histology (HR: 3.8995% CI: 1.19–12.7; *p* = 0.025, R^2^ = 0.07) and lymph node involvement (HR: 3.6395% CI: 1.71–7.71; *p* = 0.001, R^2^ = 0.08). Within this hierarchy of risk, the presence of an IVCT served as a significant, albeit comparatively more moderate, prognostic indicator (HR: 2.5395% CI: 1.12–5.73); *p* = 0.026). While an IVCT conferred a substantial increase in the hazard for an event, its overall impact on model discrimination (C-statistic = 0.58) and variance explanation (R^2^ = 0.04) placed it below the predictive weight of metastasis, nodal status, and histology (Table 3).

For OS, the univariable Cox regression analysis identified metastasis as a primary driver of mortality, demonstrating a more than five-fold risk increase (HR: 5.81; 95% CI: 2.20–15.32; *p* < 0.001) and the highest level of discrimination (C-statistic = 0.73; Nagelkerke R^2^ = 0.12). Similarly, lymph node involvement (HR: 5.73; 95% CI: 22.32–14.14; *p* < 0.001; R^2^ = 0.11) and histology (HR: 0.10; 95% CI: 0.04–0.27); *p* < 0.001; R^2^ = 0.12) were highly significant predictors of OS. Within this hierarchy of prognostic significance, the presence of an IVCT remained a significant risk factor (HR: 3.27; 95% CI: 21.23–8.67; *p* = 0.017), though its contribution to model variance (R^2^ = 0.04) and discrimination (C-statistic = 0.61) was notably lower than that of either metastatic or nodal status (Table 4).

## 5. Discussion

This single-center retrospective study evaluated the clinical characteristics and outcomes of pediatric patients diagnosed with a Wilms tumor (WT) and complicated by an inferior vena cava tumor thrombus (IVCT), with a comparison to those lacking vascular involvement. The reported prevalence of IVCT ranges from 4–10% in multicenter series and extends into the right atrium in <1–3% of children; our cohort falls above the higher end of this spectrum, reflecting potential referral bias or delayed presentation in our population [2]. An IVCT was more frequent in our patient cohort than in most literature reports, possibly due to underlying differences in tumor biology or a tendency toward advanced-stage disease at initial evaluation.

Consistent with previously published reports, children with an IVCT in our cohort were older at diagnosis and more frequently presented with advanced-stage disease, including higher rates of pulmonary metastasis, a larger tumor size, and lymph node involvement [18,19]. Clinical features such as hematuria and an abdominal mass were also more prevalent, reflecting the more extensive local tumor burden, which aligns with prior studies [7,12,20]. While these observations might suggest a more aggressive disease, the presence of an IVCT should not be interpreted solely as a marker of inherently aggressive tumor biology. Rather, the development of an IVCT is likely multifactorial. There are different hypotheses: this could be potentially influenced by aggressive biological tumor characteristics, anatomical considerations such as tumor location and venous drainage patterns, and delays in presentation or referral. This nuanced view highlights that an IVCT may represent a complex interplay of factors affecting tumor progression and clinical presentation, rather than a single prognostic indicator [20].

In one large multicenter analysis, infra-hepatic, intra-hepatic, supra-hepatic and cardiac extensions accounted for roughly 43%, 26%, 11%, and 19% of cases, respectively, illustrating the broad spectrum of vascular invasion seen in practice [21]. Our patient cohort demonstrated a similar distribution, with infra-hepatic involvement in 53.0%, retro-hepatic in 23.6%, supra-hepatic in 5.9%, and cardiac extension in 17.6%, aligning closely with previously published figures.

The preferential involvement of the right renal vein in tumor thrombus formation is well-recognized and may be attributed to its shorter length relative to the left renal vein. Supporting this, right-sided tumors accounted for 65% of IVCT cases in our series, a significantly elevated frequency compared to the distribution in patients without IVCT. This predilection has been consistently reported in multiple cohorts, with right-sided tumors accounting for 55–70% of IVCT cases [12,20].

Our findings showed that survival outcomes among patients with IVC involvement were inferior to those reported in published series, and were also lower compared with patients without vascular extension [20]. However, these differences might be driven by the higher burden of metastatic disease and advanced local stage in the IVCT group, rather than the presence of IVCT itself. Metastatic disease was present in 65% in the IVCT group compared with 19% the non-IVCT group; among the 17 patients with IVCT, 11 had stage IV disease and four had stage III disease accounting for 89% of the cases, compared to 41% in patients without IVCT. Consistent with this observation, univariable analysis showed that older age, metastatic disease, IVCT, LN involvement, and diffuse anaplasia were associated with inferior EFS and OS. While the presence of an IVCT remained a statistically significant prognostic indicator across both EFS and OS, its individual contribution to model variance (Nagelkerke R^2^ = 0.04) and discrimination (C-statistic: 0.58–0.61) was consistently lower than the predictive weight of metastasis, lymph node involvement, and high-risk histology. These findings align with cooperative group data suggesting that IVCT is primarily a marker of disease extent rather than an independent adverse prognostic factor [7,21]. Anaplasia is recognized as a major prognostic adverse histologic subtype in Wilms tumor and is associated with more aggressive disease biology and poorer outcomes. In cohorts of WT with intravascular extension, unfavorable or anaplastic histology, although less frequent than favorable histology, is linked to inferior EFS and OS, underscoring the importance of tumor biology in these presentations [22].

Regression of the tumor thrombus may reduce surgical complexity by eliminating the need for sternotomy and cardiopulmonary bypass (CPB), and/or vena cava grafting [12,20,23]. Accordingly, the use of the neo-adjuvant chemotherapy remains a cornerstone of management, as it plays a critical role in reducing tumor thrombus burden and facilitating complete surgical resection [6,12,24,25,26]. In contrast to the SIOP approach, which mandates preoperative chemotherapy for all patients with WT and IVCT [27], the NWTS/Children Oncology Group (COG) strategy is risk- and extent-adapted, emphasizing upfront surgery when feasible and selective use of neo-adjuvant chemotherapy [28,29]. Preoperative chemotherapy is explicitly recommended when tumor thrombus extends to hepatic veins and above, with the aim of reducing intraoperative complications [14]. Neo-adjuvant chemotherapy has been shown to induce tumor thrombus regression in nearly half of the affected patients, and multiple cohorts have demonstrated that complete thrombus resolution occurs most frequently in patients with infra-hepatic IVCT at diagnosis [6,8,19]. Following neo-adjuvant chemotherapy, all patients with IVCTT underwent delayed radical nephroureterectomy with lymph node sampling. Complete thrombus resolution was achieved in 41.2% of the IVCTT group, exclusively in cases of infra-hepatic extension, consistent with prior reports demonstrating thrombus regression rates ranging from 25–45% [19,21,24].

Despite the potential for complete thrombectomy, emerging evidence suggests that complete removal of the thrombus may not always be required for favorable oncologic outcomes. In a recent large cooperative group series, neither incomplete resection nor the presence of viable tumor cells within the thrombus significantly impacted EFS or OS, underscoring the effectiveness of multimodal therapy (systemic therapy, surgery, and/or radiotherapy) in achieving excellent outcomes even with advanced intravascular extension [21].

Despite the strengths of this study, including detailed clinic–pathologic characterization and long-term follow-up, several limitations must be acknowledged. The retrospective design and single-center nature of the cohort may limit generalizability. Additionally, the relatively small number of patients with an IVCT prevented the use of multivariable models to further isolate independent risk factors. Nevertheless, this study adds to the growing body of evidence supporting the prognostic significance of vascular involvement in WTs, especially from countries with limited resources.

In conclusion, an IVCT occurs in a subset of children with WTs and is associated with more advanced local disease and a higher burden of metastases. While survival outcomes are inferior in patients with an IVCT compared with those without vascular involvement, our finding, supported by a univariable analysis showing a lower explanatory power for an IVCT, suggest that vascular extension itself is likely not an independent adverse prognostic factor. The presence of metastatic disease remains the primary determinant of outcome. These findings underscore the importance of comprehensive staging, risk-adapted therapy, and multidisciplinary management for patients with an IVCT, with therapeutic decisions guided by overall disease burden rather than vascular extension alone.

## Figures and Tables

**Table 1:** Characteristics of patients and tumor in all groups.

Variable	All Patients, Number (Percentage)	Without IVCT, Number (Percentage)	With IVCT, Number (Percentage)	*p*-Value
Total Patients	110	93	17	
Age at Diagnosis Median (range)	3.8 years (0.4–15.1)	3.5 (0.4–11.3)	4.2 (2.6–15.1)	0.019
Sex				0.5
Females	62 (56%)	51 (55%)	11 (65%)	
Males	48 (44%)	42 (45%)	6 (35%)	
Laterality				0.046
Left	63 (57%)	57 (61%)	6 (35%)	
Right	47 (43%)	36 (39%)	11 (65%)	
Signs and symptoms				
Abdominal mass	82 (75%)	69 (74%)	13 (76%)	>0.9
Hematuria	23 (21%)	15 (16%)	8 (47%)	0.008
Abdominal pain	38 (35%)	32 (34%)	6 (35%)	>0.9
Hypertension	13 (12%)	12 (13%)	1 (5.9%)	0.7
Histologic Subtypes				>0.9
Favorable	79 (71.8%)	67 (72%)	12 (71%)	
Blastemal predominant	18 (16.4%)	15 (16%)	3 (18%)	
Focal anaplasia	8 (7.3%)	4 (4.3%)	1 (5.9%)	
Diffuse anaplasia	5 (4.5%)	7 (7.5%)	1 (5.9%)	
Stage				<0.001
Stage I	32 (29.1%)	32 (34%)	0 (0%)	
Stage II	25 (22.7%)	23 (25%)	2 (12%)	
Stage III	18 (16.8%)	14 (15%)	4 (24%)	
Stage IV	35 (31.5%)	24 (26%)	11 (65%)	
Maximum tumor diameter median (range) cm	12 (2.4–23.5)	11.9 (2.4–23.5)	12.7 (7.5–20)	0.01
Positive lymph nodes	20 (18%)	13 (14%)	7 (41%)	0.014
Treatment				
Surgery	110 (100%)	93	17	0.13
Upfront	16 (14.5%)	77 (83%)	0 (0%)	
Delayed	94 (85.5%)	16 (17%)	17 (100%)	
Radiation therapy	45 (40.9%)	30 (32%)	15 (88%)	<0.001
Chemotherapy	110 (100%)	93 (100%)	17 (100%)	<0.001
Two drugs	50 (45.5%)	50(54%)	0 (0%)	
Containing doxorubicin	60 (54.5%)	43 (46%)	17 (100%)	

**Table 2:** Distribution of inferior vena cava thrombus level at diagnosis compared with post-chemotherapy status.

Post-Chemotherapy Thrombus Level/ Thrombus Level at Diagnosis	Infra-Hepatic	Retro-Hepatic	Resolved	Supra-Hepatic	Percentage
Infra-hepatic	4	0	5	0	52.9%
Retro-hepatic	0	2	2	0	23.6%
Supra-hepatic	0	1	0	0	5.9%
Intra-cardiac	0	0	0	3	17.6%
Percentage	23.6%	17.6%	41.2%	17.6%	100%

**Table 3:** Univariate cox regression model and Pseudo R^2^ and discrimination for event-free survival (EFS) for all patients.

Parameter				Cox Regression		Goodness-of-Fit (Pseudo R^2^)		Discrimination	
		N	Events	HR, 95% CI	*p*-Value	Nagelkerke R^2^	Scaled R^2^	C-Statistic	SE (C)
Gender	Male vs. Female	110	29	0.74 (0.35–1.57)	0.44	0.006	0.006	0.53	0.047
Age at diagnosis	Median (3.8 year)	110	29	1.14 (1.03–1.26)	0.009	0.06	0.07	0.62	0.045
Metastasis	Yes vs. No	110	29	3.86 (1.84–8.11)	<0.001	0.11	0.12	0.67	0.045
Histology	DA vs. others	110	29	3.89 (1.19–12.7)	0.025	0.07	0.08	0.57	0.035
LN involvement	Positive vs. Negative	110	29	3.63 (1.71–7.71)	0.001	0.08	0.09	0.63	0.044
IVCT	Yes vs. No	110	29	2.53 (1.12–5.73)	0.026	0.04	0.04	0.58	0.04

Abbreviations: HR, hazards ratio; CI, confidence interval; SE, standard error; IVCT, inferior vena cava thrombus; DA, diffuse anaplasia; LN, lymph node.

**Table 4:** Univariable Cox regression and Pseudo R^2^ and discrimination for overall survival (OS) for all patients.

Parameter				Cox Regression		Goodness-of-Fit (Pseudo R^2^)		Discrimination	
		N	Events	HR, 95% CI	*p*-Value	Nagelkerke R^2^	Scaled R^2^	C-Statistic	SE (C)
**Gender**	**Male vs. Female**	110	19	0.92 (0.37–2.29)	0.86	0.00	0.000	0.51	0.06
**Age at diagnosis**	**Median** **(3.8 year)**	110	19	2.5 (0.98–6.38)	0.055	0.034	0.045	0.60	0.059
**Metastasis**	**Yes vs. No**	110	19	5.81 (2.20–15.32)	<0.001	0.12	0.12	0.73	0.051
**Histology**	**DA vs. others**	110	19	0.10 (0.04–0.27)	<0.001	0.12	0.16	0.64	0.055
**LN involvement**	**Positive vs. Negative**	110	19	5.73 (2.32–14.14)	<0.001	0.11	0.15	0.70	0.058
**IVCT**	**Yes vs. No**	110	19	3.27 (1.23–8.67)	0.017	0.04	0.055	0.61	0.055

Abbreviations: HR, hazards ratio; CI, confidence interval; SE, standard error; LN, lymph node; IVCT, inferior vena cava thrombus.

## Data Availability

The data presented in this study are available on request from the corresponding author due to ethical restrictions.

## Abbreviations

CI	Confidence Interval
CPB	Cardiopulmonary Bypass
CT	Computed Topography
COG	Children Oncology Group
EFS	Event-Free Survival
HR	Hazard Ratio
IVCT	Inferior Vena Cava Thrombus
KHCC	King Hussein Cancer Center
MDT	Multidisciplinary Team
MRI	Magnetic Resonance Imaging
OS	Overall Survival
NWTS	National Wilms Tumor Study
SIOP	International Society of Pediatric Oncology
VA	Vincristine Actinomycin D
WT	Wilms Tumor
